# Reducing hemolysis in emergency departments: effectiveness of BD Barricor™ low-vacuum tubes

**DOI:** 10.1515/almed-2025-0185

**Published:** 2026-04-23

**Authors:** Berta Sufrate-Vergara, Daniel Prieto Arribas, Isabel Moreno-Parro, Jorge Díaz-Garzón Marco, Roberto Mora Corcovado, Enrique Torres Rodríguez, Alberto Iglesias Sigüenza, Ana María Souto Novas, Antonio Buño Soto, Pilar Fernández-Calle

**Affiliations:** Laboratory Medicine Department, 16268 La Paz University Hospital, Madrid, Spain; 16268 Health Research Institute of La Paz University Hospital (IdiPAZ), Madrid, Spain; Emergency Department, 16268 La Paz University Hospital, Madrid, Spain

**Keywords:** hemolysis, emergency department, low-vacuum tubes, Barricor^TM^

## Abstract

**Objectives:**

Hemolysis is the leading cause of sample rejection in laboratories, particularly in emergency departments (EDs), where rates often exceed recommended limits. Hemolysis not only delays diagnosis and treatment but also increases costs. Despite protocol reinforcement and staff training, in our tertiary hospital’s ED we experienced consistently high hemolysis rates, meaning a high percentage of results were not reported to clinicians due to the interference. Low-vacuum tubes, such as BD Barricor™ (that has a mechanical separator), may reduce hemolysis and improve sample quality.

**Methods:**

We conducted a single-centre prospective pilot study over 1 week substituting standard lithium-heparin tubes with BD Barricor™ tubes in the ED. Sample handling, transportation, centrifugation, and analysis remained unchanged. Hemolytic index (HI), potassium, LDH, AST, creatinine and phosphate results were extracted from the laboratory information system for the trial week and 2 weeks before and after. Statistical analysis was performed with R (v.4.2.3).

**Results:**

During the trial week, median HI decreased a 28 % (p<0.05). Samples exceeding established HI thresholds dropped from 16–19 % to <6 % (p<0.05). The proportion of unreported tests fell from an average of 250 per week to 43 (0.8 %) during the trial week. The use of Barricor™ tubes enabled nearly all results to be reported.

**Conclusions:**

Implementing low-vacuum Barricor™ tubes in our ED significantly reduced hemolysis and decreased non-reported tests. Our findings suggest that implementing technological solutions may be hihgly effective when protocol reinforcement alone did not work. Long-term studies are warranted to assess cost-effectiveness and broader clinical impact.

## Introduction

Hemolysis is the most common cause of sample rejection in the laboratory. It accounts for 40–70 % of the rejected samples, significantly exceeding other pre-analytical causes [1], [2], [3]. *In vitro* hemolysis occurs during the pre-analytical phase, a complex phase involving many steps such as blood extraction or sample transportation [4]. Most of these steps take place outside the laboratory and many of them are still manual and operator dependent, making it the stage of the total testing process in which most errors occur. Pre-analytical errors affect the quality of the sample and the results later issued by the laboratory [5].

In addition to the disruption to the patient and the delay in diagnosis/treatment, sample rejections due to hemolysis have an economic impact [6], 7]. In 2021, Phelan et al. [7] estimated the direct cost of samples being rejected due to hemolysis in an ED with a 10 % hemolysis incidence, demonstrating the huge economic impact and expense associated to these type of rejections.

One of the key quality indicators that we use in the laboratory to monitor and improve the pre-analytical phase is related to hemolysis and is defined as the percentage of test requests with at least one result not reported due to a high degree of hemolysis interfering the measurement.

La Paz University Hospital is a tertiary care centre located in Madrid, Spain, serving a population of more than 550,000 people. The Emergency Department (ED) treats an average of 515 adult patients per day with peaks of more than 800 patients in the same day. The 24-h/emergency laboratory of the Laboratory Medicine Department receives an average of 600 urgent requests per day, with half of them coming from the ED.

It is well known that hemolysis rates are higher in EDs due to patients being in worse conditions, extractions are more difficult, etc. [8]. However, in our experience, hemolysis data from the ED has consistently and significantly exceeded the established 7 % limit based on literature [4], 8], 9]; in fact, the number of rejected tests has been over the 10 % every month for the last 4 years. This high percentage of rejections due to hemolysis results in unreported tests to the requesting clinician and the need to repeat the patient’s analysis, causing inconvenience, extra costs and delays in diagnosis and patient management.

We do not see this persistent problem with any other department in the hospital, as actions have been effectively performed when higher hemolysis percentages were observed. The relevance of the problem in terms of the amount of tests requested but not informed due to the interference has been clearly and persistently communicated to the ED professionals. Additionally, protocols following EFLM guidelines [10], 11] to avoid hemolysis when collecting blood have been reinforced. Despite these efforts over time, we have not seen significant changes in the hemolysis indicator. It is noteworthy that, in addition to the particular and complex characteristics of ED’s patients, our ED has a very large number of nursing staff with a heavy patient load and suffers from constant turnovers and relocations, making it harder for the information and seriousness of the issue to reach all the constantly changing personnel.

There is evidence suggesting that partial draw vacuum blood collection tubes decrease hemolysis rates [12], [13], [14], [15], [16]. Becton Dickinson, USA (BD) has developed a lithium heparin tube with reduced vacuum named BD Barricor™. Additionally, the tube uses a mechanical separator instead of gel, providing a more stable plasma sample with no gel residues or fibrin after centrifugation.

Given that other ways to reduce hemolysis in the ED such as reviewing protocols and raising staff awareness of the problem had already been explored without seeing significant improvement, we decided to explore the possibility and evaluate the use of BD Barricor™ low-vacuum tubes as a potential solution to our persistent hemolysis issues.

The primary aim of this study was to compare hemolysis rates and test rejection frequency between the conventional lithium-heparin tubes and the BD Barricor™ tubes in the adult Emergency Department in a tertiary hospital.

## Materials and methods

We conducted a single-centre prospective study to test Barricor™ tubes to see if we could effectively reduce the incidence of hemolyzed samples in the ED. This was a pilot study carried out for just 1 week with the aim of representing a sufficient period that included all consecutive days of the week.

During the week of May 5 to May 11, 2025 (inclusive), the standard lithium-heparin tubes (Kima, Italy), routinely used in the ED of La Paz University Hospital, were substituted by BD Barricor™ tubes. Before introducing these new tubes, nursing staff were informed that, due to their lower vacuum, Barricor™ tubes would require a slightly longer filling time during sample collection. Apart from this adjustment, nurses were instructed to maintain all other aspects of their standard collection procedure unchanged.

The post-collection sample handling process remained identical to that used during any other week utilizing standard lithium-heparin tubes. This included sample transportation via pneumatic tube system to the emergency laboratory, centrifugation, and subsequent processing through the automated analytical system. Although existing literature suggests that Barricor™ tubes allow shorter centrifugation times to obtain plasma, we applied the same centrifugation protocol to both tube types – 3,000 *g* for 10 min – and no other intervention took place in the following steps of the analysis procedure to ensure consistency in the comparison.

Data from the 2 weeks prior to the trial (weeks 17 and 18), the trial week itself (week 19), and the two following weeks (weeks 20 and 21) was extracted from the laboratory information system (LIS) (Servolab; Siemens Healthineers, Germany).

For the comparison, we decided to collect results from those analytes whose concentrations are more affected by the presence of hemolysis, so we chose potassium, lactate dehydrogenase (LDH), aspartate aminotransferase (AST), creatinine and phosphate. We also gathered the measured hemolytic index (HI) results, expressed as mg/dL of free Hb.

Samples were analysed in the Atellica Solution analyzer (Siemens Healthineers, Germany).

Beyond the information provided by the provider in its instructions for use (IFU), cut-off points based on experimental interferograms have been established in our laboratory to assess whether the results obtained for a given measurand are not interfered and can be reported to the clinician or the sample is so hemolyzed that it interferes with the measurement. For LDH, we do not report results when the hemolytic index (HI) exceeds 100 mg/dL; for potassium and AST, when HI exceeds 200 mg/dL; and, for creatinine, the cut-off is 500 mg/dL. In the case of phosphate, results are not suppressed, however, a comment is added to inform the clinician of possible overestimation due to the HI being above 500 mg/dL.

Statistical analysis and graphic representation were performed with R (v.4.2.3)/RStudio (v.2023.09.01) using ggplot2 (v.3.5.0) and Tidyverse (v.2.0.0) packages. The normal distribution of the different variables was tested using the Shapiro-Wilk test. To determine whether the differences between groups were statistically significant, the Mann-Whitney and Kruskal-Wallis tests were applied.

## Results

A total of 34,270 results from 8,474 episodes corresponding to 6,581 different patients were extracted from the LIS for the 5 weeks above mentioned. The number of episodes ordered from the ED during the 5-week span was very similar, with an average of 1,700 requests per week (242 per day). Creatinine, potassium and AST were the most demanded tests (8,372, 8,351 and 5,877 times, respectively). More detailed information on number of requests by week is shown in Supplementary Table 1.

**Figure 1:**
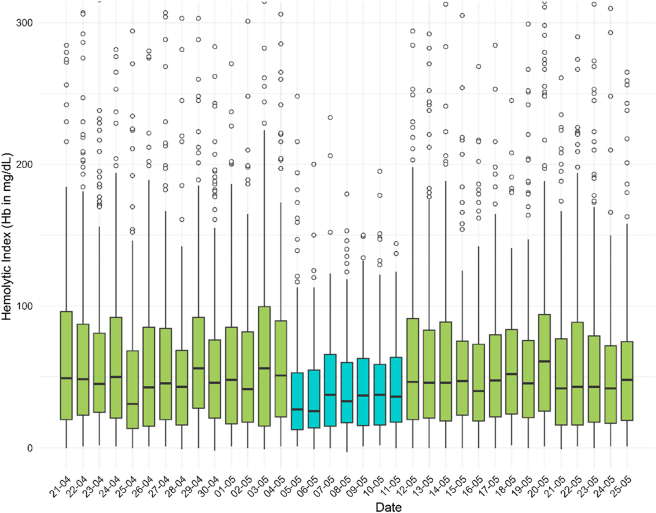
Boxplot representation of the hemolytic index results per day. Represented in a different colour (blue) the week of the pilot trial.

The median HI during week 19 (the week of the trial) was 33 (IQR: 44) mg/dL, whereas the other 4 weeks it was 46 (IQR: 65) mg/dL. This meant a statistically significant (p<0.001) decrease of 28 % during the trial week. Dispersion, as shown with the IQR, was also lower during week 19. We also observed significant differences when comparing the HI of each individual week with that of the week in which the Barricor™ tube was used. HI results per day and per week are presented in Figure 1 and Supplementary Table 2, respectively.

**Figure 2:**
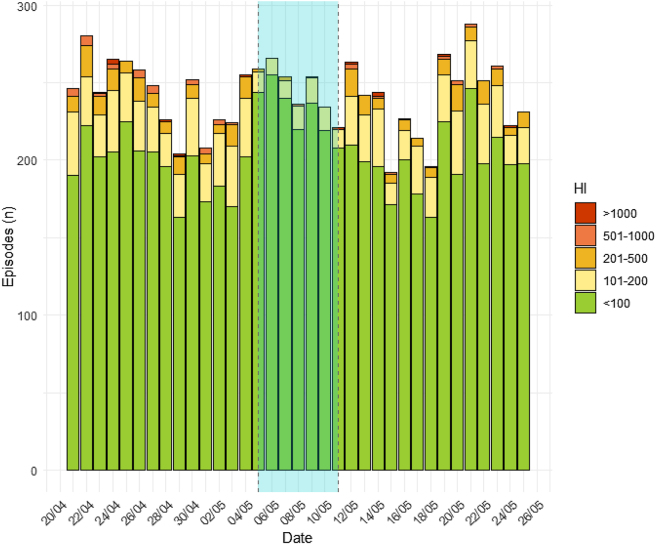
Number of episodes with HI>100 ordered by HI range and date. In blue, week 19 (the week of the Barricor™ trial).

The total number of samples with HI above 100 mg/dL dropped by nearly two-thirds in week 19, going from representing 16–19 % of all episodes in weeks 17, 18, 20, and 21 to less than 6 % during that week. Results broken down by HI range by week and day are available in Table 1 and Figure 2, respectively. Statistically significant differences were observed when comparing the number of requests for each HI range the trial week with the others.

**Table 1:** Number of episodes and percentage in relation to the total number of tests ordered per week by hemolytic index (HI) range.

Week	HI < 100	HI 101–200	HI 201–500	HI 501–1,000	HI > 1,000
17	1,455 (80.6 %)	232 (12.9 %)	88 (4.8 %)	26 (1.4 %)	4 (0.2 %)
18	1,290 (80.9 %)	222 (13.9 %)	68 (4.2 %)	12 (0.8 %)	3 (0.2 %)
19	1,623 (94.1 %)	93 (5.4 %)	5 (0.3 %)	1 (0.1 %)	2 (0.1 %)
20	1,317 (83.5 %)	188 (11.8 %)	62 (3.9 %)	7 (0.4 %)	4 (0.3 %)
21	1,470 (83.0 %)	215 (12.1 %)	77 (4.3 %)	8 (0.4 %)	2 (0.1 %)

**Figure 3:**
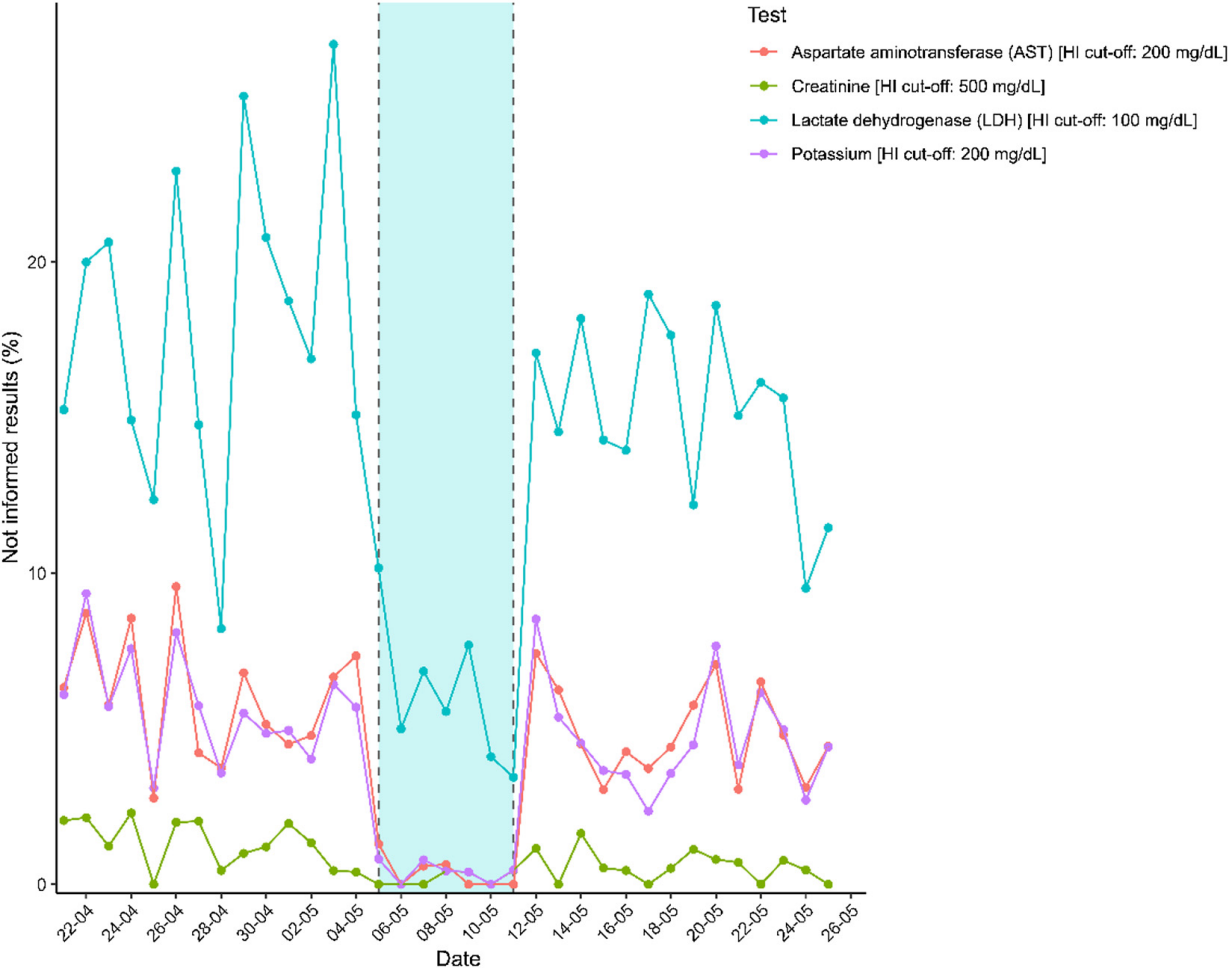
Percentage of not informed results due to hemolysis per day.

During week 19, only 43 (0.8 %) of the 5,125 results were not reported due to the HI being over the established limits (29 LDHs, 7 potassiums, 4 ASTs and 3 creatinines), compared to an average of 250 tests erased per week during any of the other weeks. To put this data into perspective, in just 1 day during week 17, 48 results (26 potassium and 22 LDH) were not informed due to hemolysis. Table 2 shows the number of unreported tests per week and the percentage of the total they represented. In addition, Figure 3 illustrates the percentage of unreported results per test and per day. LDH was the test that had to be deleted most often, with a maximum of 27.0 % not reported tests on 03/05/2025, week 18. However, the maximum in week 19 was 7.7 % on 09/05/2025.

**Table 2:** Number of deleted tests and percentage per test and week.

Week	LDH	Potassium	AST	Creatinine	Total
17	91 (17.3 %)	117 (6.6 %)	84 (6.6 %)	30 (1.7 %)	322 (6.0 %)
18	88 (18.9 %)	78 (5.0 %)	64 (5.6 %)	15 (1.0 %)	245 (5.2 %)
19	29 (5.9 %)	7 (0.4 %)	4 (0.3 %)	3 (0.2 %)	43 (0.9 %)
20	77 (16.3 %)	73 (4.7 %)	51 (4.9 %)	10 (0.6 %)	211 (4.6 %)
21	81 (14.0 %)	86 (4.9 %)	63 (5.0 %)	10 (0.6 %)	240 (4.5 %)

We have compared the number of episodes with concentrations above the higher limit of the reference intervals (RI) and, among these, how many of them were finally not reported due to the samples being highly hemolyzed. A reduction during week 19 for potassium and AST was observed, however, this was not the case for LDH. The number of phosphate results above the RI remained similar over the 5 weeks (between four and six results per week). When it comes to creatinine, the effect of hemolysis is the opposite, causing an underestimation of the concentration. The percentage of not informed results in the aforementioned episodes was significantly lower in week 19 in comparison to the other 4 weeks. Data is presented in Table 3.

**Table 3:** Number of results above the RI and number of those results not informed due to hemolysis. RIs applied in our laboratory: potassium: 3.5–5.1 mmol/L; LDH: 100–246 IU/L; AST: <40 IU/L; creatinine: 0.7–1.3 mg/dL.

Week	Potassium			LDH			AST			Creatinine		
	Total	> RI	Not informed	Total	> RI	Not informed	Total	> RI	Not informed	Total	< RI	Not informed
17	1,782	127 (7.1 %)	75 (59.0 %)	527	290 (55.1 %)	91 (31.4 %)	1,269	282 (22.2 %)	66 (23.4 %)	1,783	627 (35.2 %)	17 (2.7 %)
18	1,559	96 (6.1 %)	43 (44.8 %)	466	267 (57.3 %)	88 (32.9 %)	1,139	251 (22.0 %)	47 (18.7 %)	1,577	599 (38.0 %)	8 (1.3 %)
**19**	**1,691**	**45 (2.7 %)**	**3 (6.7 %)**	**491**	**346 (70.5 %)**	**29 (8.4 %)**	**1,170**	**216 (18.5 %)**	**4 (1.9 %)**	**1,702**	**614 (36.1 %)**	**3 (0.5 %)**
20	1,567	89 (5.7 %)	43 (48.3 %)	472	249 (52.8 %)	77 (30.9 %)	1,032	229 (22.2 %)	40 (17.5 %)	1,560	575 (36.9 %)	6 (1.0 %)
21	1,752	92 (5.3 %)	44 (47.8 %)	579	301 (51.9 %)	81 (26.9 %)	1,267	282 (22.3 %)	56 (19.9 %)	1,750	636 (36.3 %)	5 (0.8 %)

Bold values indicate results from the trial week (week 19).

We also compared hypokalemia over the 5 weeks, as the hemolysis of the sample could be causing falsely within range potassium concentrations to be reported in patients that actually have levels below the RI. However, the percentage of results below 3.5 mmol/L remained constant over the 5 weeks (around 7 %) and no significant differences were observed when comparing weeks.

Supplementary Table 3 shows in more detail the percentage of results for each parameter classified according to whether they were below, above or within the RIs, and how many of these results were deleted due to the established HI cut-offs.

As mentioned in M&M, in the case of phosphate, the results are not eliminated, but rather an explanatory comment is added when the HI is greater than 500 mg/dL. During the week of the trial, there were no phosphate determinations in samples with HI>500 mg/dL, while in previous weeks the comment had to be added eight times (five times in week 17 and once a week in weeks 18, 20 and 21). Additionally, only one of those eight phosphates was above the established RI (2.4–5.1 mg/dL).

## Discussion

This study evaluated the effect of using low-vacuum lithium heparin Barricor™ tubes with a mechanical separator as a measure to reduce the incidence of hemolysis in the ED and its repercussion on laboratory results.

In line with what we have been observing for years and our long-term results, during the 4 weeks in which the Barricor™ tube was not used, the percentage of LDH rejections (that has the lowest HI cuttoff of the tests evaluated) was between 18.9 and 14.0 % depending on the week. This percentage decreased significantly to 5.9 % in the week using the Barricor™ tube. A marked decrease was also observed in the rest of the tests, since, as can be seen in Table 1, the number of tests with HI above 200 also decreased from 6.5 to 0.5 %.

During the trial week, only 101 of 1,724 processed samples showed HI values above 100 mg/dL – the threshold for LDH suppression – and only eight surpassed 200 mg/dL, the threshold cutoff for potassium and AST. As expected, the highest percentage of deleted results corresponds to LDH, as it is the most affected analyte by hemolysis, with a maximum of almost a third of the requested determinations being deleted on one day (27.0 % on 03/05/2025, week 18). Although during the test week it also remained by far the most unreported test, the maximum of deleted tests was 7.7 % on 09/05/2025. Notably, nearly 99 % of potassium, AST, and creatinine results were successfully reported, reinforcing the clinical utility of the Barricor™ tubes. Badiou et al. [15] also found a statistically significant decrease in the percentage of potassiums and AST results with HI above their cutoff (90 and 40 mg/dL of free Hb, respectively).

In accordance with literature [17], our findings show that hemolysis interference affects LDH, potassium, and AST causing falsely elevated results while creatinine results are decreased. Although hemolyzed results are excluded from clinical reporting, the values are retained within the LIS, allowing us to compare the frequency of elevated values across weeks (data shown in Table 3). In week 19 (the trial week), episodes with elevated potassium and AST levels decreased, as did the proportion of those results that were suppressed due to hemolysis. Creatinine results below the RI stayed similar during the 5 weeks, but the percentage of those results deleted due to high HI significantly decreased. For LDH, while the proportion of hemolysis-related suppression also decreased, there was a paradoxical increase in the number of patients with elevated LDH concentrations. Nevertheless, during the trial week, a greater proportion of elevated LDH results were reportable, enabling their use in clinical decision-making for critically ill patients and avoiding the need to re-puncture the patient or make decisions without knowing all the information requested in the analysis.

We did not identify any factors that would explain a causal relationship between the use of Barricor™ tubes and the increase in LDH values; this trend appears random and may warrant further exploration, especially considering the lack of diagnostic information in this study design. Median concentrations compared take into account all measurements made during the study period, including results that were ultimately not reported because they were considered to be interfered by hemolysis, so the decrease in erased data does not explain the difference in median concentrations. Reviewing literature, there are discrepancies regarding the variation in LDH concentration, although most studies report decreases in it. Raizman et al. [18] also found higher LDH values in the determinations made with the Barricor™ tube. He hypothesises that, when using a tube with separator gel, the barrier between plasma and cell content is quickly formed and stays almost impenetrable; however, the mechanical separator in the Barricor™ tube is always slightly open and allows some of the cellular content to pass through, which could explain the increase in LDH. Nevertheless, this effect was not observed in any other measurand also affected by hemolysis and the release of cellular contents, although it is true that LDH is the most sensitive to these effects.

Many articles [19], [20], [21], [22], [23] comparing Barricor™ with other plasma or serum tubes report potassium, AST, and phosphate concentrations decrease in the former, as we have seen in our study. Aksit et al. [24] report slightly higher concentrations of potassium in the Barricor™ vs. the lithium heparine tube. As mentioned before in this discussion, only Badiou [15] relates these results with HI levels also decreasing.

We consider that our study is innovative and different from previous publications on this type of tube due to its design and approach. Most studies on Barricor™ compare it with serum tubes with separating gel, and very few compare it with lithium heparine plasma ones. Furthermore, the variables they evaluate are generally turnaround time reduction (due to the need for much less centrifugation time) and increased stability/improved storage times [15], 23], 25]. Although it is not the main objective of any of these studies and is not presented as such, several articles report decreased hemolysis’ levels in Barricor™. In more general terms, and without referring to this specific tube, there is literature [12], [13], [14], [15] stating that the use of low-vacuum tubes reduces hemolysis levels, in line with the findings of our study.

As for the limitations of the study, we consider them to be the limited 1-week duration, the fact that it was conducted in a single location (ED) – albeit the one with the highest and most persistent hemolysis rates, and that it has not been possible to compare the results obtained with clinical data to assess the impact on patients’ outcomes.

Although it was not the primary aim of the study and complete data to estimate this impact are lacking, potential savings associated with the use of the Barricor™ should be considered not only in terms of patient discomfort or delays in diagnosis and treatment, as previously noted, but also in material resources (needles, tubes) and personnel required for repeated analysis [7].

In our opinion, the 1-week trial using BD Barricor™ tubes yielded highly satisfactory results. Hemolysis levels decreased significantly, as did the number of samples exceeding critical HI thresholds, which led to a marked reduction in non-reported test results. Our findings suggest that, in a high-turnover environment like our ED, where protocol reinforcement has proven insufficient, implementing a technological, system-level solution like Barricor™ tubes is a highly effective strategy to reduce hemolysis-related rejections and improve laboratory efficiency. Future studies should evaluate long-term implementation and cost-effectiveness in routine ED workflows and other environments.

## Supplementary Material

Supplementary Material
