# Supplementary material for: Reducing hemolysis in emergency departments: effectiveness of BD Barricor™ low-vacuum tubes
Source: Adv Lab Med. 2026 Apr 23;7(2):116–23. doi: 10.1515/almed-2025-0185 (PMC13169830; doi:10.1515/almed-2025-0185)
Supplement: Supplementary file 1 — Supplementary Material [file j_almed-2025-0185_suppl_001.docx]

**SUPPLEMENTARY MATERIAL: 3 TABLES**

**Supplementary Table 1.** Number of tests ordered per week.

| **Week** | **Creatinine** | **Potassium** | **AST** | **LDH** | **Phosphate** | **Hemolytic index (HI)** |
| --- | --- | --- | --- | --- | --- | --- |
| **17** | 1,783 | 1,782 | 1,269 | 527 | 132 | 1,805 |
| **18** | 1,577 | 1,559 | 1,139 | 466 | 113 | 1,595 |
| **19** | 1,702 | 1,691 | 1,170 | 491 | 111 | 1,724 |
| **20** | 1,560 | 1,567 | 1,032 | 472 | 88 | 1,578 |
| **21** | 1,750 | 1,752 | 1,267 | 579 | 149 | 1,772 |

**Supplementary Table 2.** Median hemolytic index, interquartile range (IQR) and number of tests performed during the five weeks of the study. Medians showing statistically significant differences from week 19 are marked in bold and p-values are presented.

| **Week** | **Tests (n)** | **Median (mg/dL)** | **IQR (mg/dL)** | **p-value** |
| --- | --- | --- | --- | --- |
| 17 | 1,805 | **45** | 68 | <0.001 |
| 18 | 1,595 | **49** | 66 | <0.001 |
| 19 | 1,724 | 33 | 44 |  |
| 20 | 1,578 | **46** | 63 | <0.001 |
| 21 | 1,772 | **46** | 62 | <0.001 |

**Supplementary table 3.** Number of results with concentrations below, above, and within the reference interval (RI) and number of these that have been deleted according to HI cut-offs. RIs applied in our laboratory: potassium: 3.5-5.1 mmol/L; LDH: 100-246 IU/L; AST: <40 IU/L; creatinine: 0.7-1.3 mg/dL.

|  |  | **Potassium** | | **LDH** | | **AST** | | **Creatinine** | |
| --- | --- | --- | --- | --- | --- | --- | --- | --- | --- |
| **Week** |  | **Total** | **Not informed** | **Total** | **Not informed** | **Total** | **Not informed** | **Total** | **Not informed** |
| 17 | < RI | 104  (5.8%) | 0  (0.0%) | 0  (0.0%) |  | 0  (0.0%) |  | 627  (35.2%) | 17  (2.7%) |
|  | RI | 1551  (87.0%) | 42  (2.7%) | 237  (45.0%) | 0  (0.0%) | 987  (77.8%) | 18  (1.8%) | 966  (54.2%) | 10  (1.0%) |
|  | > RI | 127  (7.1%) | 75  (59.1%) | 290  (55.0%) | 91  (31.4%) | 282  (22.2%) | 66  (23.4%) | 190  (10.7%) | 3  (1.6%) |
| 18 | < RI | 111  (7.1%) | 0  (0.0%) | 2  (0.4%) | 0  (0.0%) | 0  (0.0%) |  | 599  (38.0%) | 8  (1.3%) |
|  | RI | 1352  (86.7%) | 35  (2.6%) | 197  (42.3%) | 0  (0.0%) | 888  (78.0%) | 17  (1.9%) | 806  (51.1%) | 6  (0.7%) |
|  | > RI | 96  (6.2%) | 43  (44.8%) | 267  (57.3%) | 88  (33.0%) | 251  (22.0%) | 47  (18.7%) | 172  (10.9%) | 1  (0.6%) |
| 19 | < RI | 114  (6.7%) | 0  (0.0%) | 1  (0.2%) | 0  (0.0%) | 0  (0.0%) |  | 614  (36.1%) | 3  (0.5%) |
|  | RI | 1532  (90.6%) | 4  (0.3%) | 144  (29.3%) | 0  (0.0%) | 954  (81.5%) | 0  (0.0%) | 902  (53.0%) | 0  (0.0%) |
|  | > RI | 45  (2.7%) | 3  (6.7%) | 346  (70.5%) | 29  (8.4%) | 216  (18.5%) | 4  (1.9%) | 186  (10.9%) | 0  (0.0%) |
| 20 | < RI | 108  (6.9%) | 1  (0.9%) | 0  (0.0%) |  | 0  (0.0%) |  | 575  (36.9%) | 6  (1.0%) |
|  | RI | 1370  (87.4%) | 29  (2.1%) | 223  (47.2%) | 0  (0.0%) | 803  (77.8%) | 11  (1.4%) | 819  (52.5%) | 3  (0.4%) |
|  | > RI | 89  (5.7%) | 43  (48.3%) | 249  (52.8%) | 77  (30.9%) | 229  (22.2%) | 40  (17.5%) | 166  (10.6%) | 1  (0.6%) |
| 21 | < RI | 147  (8.4%) | 0  (0.0%) | 0  (0.0%) |  | 0  (0.0%) |  | 636  (36.3%) | 5  (0.8%) |
|  | RI | 1513  (86.4%) | 42  (2.8%) | 278  (48.0%) | 0  (0.0%) | 985  (77.7%) | 7  (0.7%) | 931  (53.2%) | 5  (0.5%) |
|  | > RI | 92  (5.3%) | 44  (47.8%) | 301  (52.0%) | 81  (26.9%) | 282  (22.3%) | 56  (19.9%) | 183  (10.5%) | 0  (0.0%) |
